# CRISPR/Cas13a-assisted amplification-free miRNA biosensor *via* dark-field imaging and magnetic gold nanoparticles†

**DOI:** 10.1039/d4sd00081a

**Published:** 2024-07-11

**Authors:** Jae-Jun Kim, Jae-Sang Hong, Hyunho Kim, Moonhyun Choi, Ursula Winter, Hakho Lee, Hyungsoon Im

**Affiliations:** a Center for Systems Biology, Massachusetts General Hospital Boston MA 02114 USA im.hyungsoon@mgh.harvard.edu +1 617 643 5679; b Department of Radiology, Massachusetts General Hospital Boston MA 02114 USA

## Abstract

MicroRNAs (miRNAs) are short (about 18–24 nucleotides) non-coding RNAs and have emerged as potential biomarkers for various diseases, including cancers. Due to their short lengths, the specificity often becomes an issue in conventional amplification-based methods. Next-generation sequencing techniques could be an alternative, but the long analysis time and expensive costs make them less suitable for routine clinical diagnosis. Therefore, it is essential to develop a rapid, selective, and accurate miRNA detection assay using a simple, affordable system. In this work, we report a CRISPR/Cas13a-based miRNA biosensing using point-of-care dark-field (DF) imaging. We utilized magnetic-gold nanoparticle (MGNPs) complexes as signal probes, which consist of 200 nm-sized magnetic beads and 60 nm-sized gold nanoparticles (AuNPs) linked by DNA hybridization. Once the CRISPR/Cas13a system recognized the target miRNAs (miR-21-5p), the activated Cas13a cleaved the bridge linker containing RNA sequences, releasing 60 nm-AuNPs detected and quantified by a portable DF imaging system. The combination of CRISPR/Cas13a, MGNPs, and DF imaging demonstrated amplification-free detection of miR-21-5p within 30 min at a detection limit of 500 attomoles (25 pM) and with single-base specificity. The CRISPR/Cas13a-assisted MGNP-DF assay achieved rapid, selective, and accurate detection of miRNAs with simple equipment, thus providing a potential application for cancer diagnosis.

## Abstract 2

CRISPR/Cas13a-based rapid amplification-free miRNA detection using a portable dark-field imaging system in 30 min.

## Graphical abstract

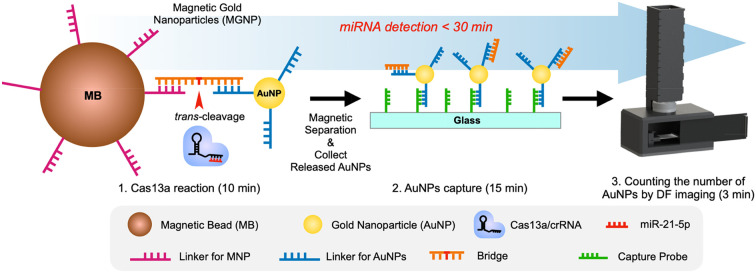

## Introduction

MicroRNAs (miRNAs) are a class of small non-coding RNAs, typically 18–24 nucleotides in length, which exert negative regulation on gene expression.^1^ They play pivotal roles in numerous cellular processes,^2^ and aberrant miRNA expression has been implicated in various human cancers, including breast, lung, pancreatic, gastric, and colorectal cancers.^3–12^ In addition, several miRNAs have been reported as potential biomarkers for early cancer detection and prognosis.^4,5,8,9,12^

However, the detection and analysis of miRNAs pose significant technical challenges. Amplification-based methods, such as reverse transcription-quantitative polymerase chain reaction (RT-qPCR),^13,14^ often encounter difficulties in primer design due to the short length of target miRNAs. While effective, hybridization-based techniques like Northern blots and microarrays are less feasible for clinical use due to their requirement for large sample volumes. Next-generation sequencing offers the advantage of simultaneously detecting multiple miRNAs and identifying novel ones. However, complex sample preparation, long assay times, and expensive operational costs hinder its application in clinical settings. Moreover, these conventional methods are primarily suitable for well-equipped laboratory environments rather than point-of-care testing.

Clustered regularly interspaced short palindromic repeats (CRISPR) and CRISPR-associated (Cas) systems have recently gained great attention for nucleic acid detection because of their advantages, such as rapid and direct detection, superior specificity (single-base mismatch), and isothermal reaction. Moreover, the CRISPR/Cas system showed attomolar sensitivity when the target RNAs were amplified by recombinase polymerase amplification (RPA).^15,16^ Although the target amplification step can increase the sensitivity, this step could introduce false-negative or false-positive results due to short lengths of miRNAs and increase the assay time. For these reasons, researchers have developed target amplification-free methods to detect miRNAs using the CRISPR/Cas system.^17–26^ The methods used three main readout techniques: fluorescence,^20,21,23,27^ colometry,^24,26^ and electrochemistry.^17–19,22,25^ Among these readout techniques, fluorescence readout is one of the most widely used techniques. Recently, droplet-digital Cas13a assay using fluorescence signal demonstrated attomolar sensitivity without target amplification.^23^ On the other hand, detecting fluorescent signals usually requires bulk and expensive equipment. Detecting the signal using colorimetry is an attractive way because the signal can be measured by the naked eye. Even though the signal can be easily measured by colorimetry, this readout is difficult to quantify accurately. Electrochemical sensors are relatively simple and cost-effective but require frequent calibration due to their instability. Thus, it is essential to develop a simple, stable, quantifiable, and cost-effective readout technique for CRISPR/Cas-based miRNA biosensors.

Here, we report a CRISPR/Cas13a-assisted miRNA detection system *via* magnetic-gold nanoparticles (MGNPs) and dark-field (DF) imaging. We started with MGNP hybrids comprising 200 nm-sized magnetic beads and 60 nm-sized gold nanoparticles (AuNPs) conjugated by DNA/RNA linkers. In the presence of target RNAs, guide RNAs recognize and hybridize to the target. The hybridization between target miRNA and guild RNA activates CRISPR/Cas13a proteins, which cleave the bridge linkers, dissociating AuNPs from magnetic beads. We developed a portable DF imaging system to accurately quantify the released AuNPs. The total assay is finished within 30 min by a simple and short reaction time of CRISPR/Cas13a (10 min), AuNPs conjugation on a glass slide (15 min), and DF imaging (3 min). This integrated approach of CRISPR/Cas13a, MGNPs, and DF imaging enables direct detection of miR-21-5p with single-base specificity and limits of detection (LOD) in the 500 attomole (25 pM). Cross-validation of assay accuracy was performed using reverse transcription-quantitative polymerase chain reaction (RT-qPCR) with small RNAs from five different breast cancer cell lines. The CRISPR/Cas13a-assisted MGNP-DF assay demonstrates rapid, selective, and accurate detection of miRNAs using simple equipment, suggesting its potential for early tumor diagnosis.

## Results and discussion

### System overview of the CRISPR/Cas13a-assisted MGNP-DF assay

**Fig. 1:**
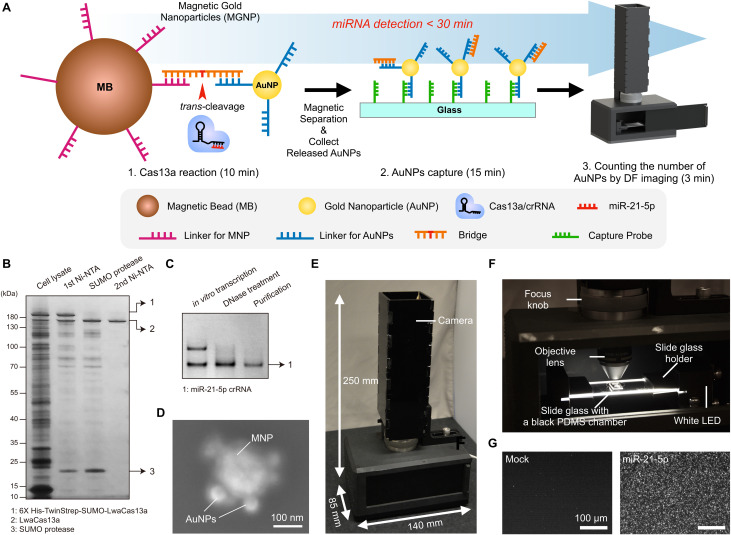
CRISPR/Cas13a-assisted magnetic nanoparticle (MGNP)-dark-field (DF) assay. A. Schematic diagram for the CRISPR/Cas13a-assisted MGNP-DF assay. In the presence of target miR-21-5p, the hybridization of the crRNA to miR-21-5p activates Cas13a proteins. Activated Cas13a proteins then cleave the bridge linkers between magnetic beads and gold nanoparticles (AuNPs). Subsequent magnetic separation of the magnetic beads releases AuNPs. These released AuNPs are captured onto a glass slide with black PDMS wells. The number of released AuNPs is quantified using a custom-built, portable DF imaging system. The entire assay can be completed within 30 min. B. Bacterial expression and purification of LwaCas13a. Purified LwaCas13a was shown by SDS-PAGE, followed by silver staining. C. Synthesis of miR-21-5p crRNA by *in vitro* transcription was confirmed by denaturing PAGE and SYBR gold staining. D. Scanning electron micrograph (SEM) of MGNP. Scale bar, 100 nm. E. Photograph of the portable DF imaging device. The overall size is 140 mm (*L*) × 85 mm (*W*) × 250 mm (*H*). F. 10× objective lens and slide glass holders coupled with a white LED. G. Representative zoomed-in DF images of AuNPs detached from magnetic beads by mock (left) or miR-21-5p treatment (right). Scale bar, 100 μm.

In the assay design, we focused on simplicity, rapid analysis, accurate quantification, and point-of-care operation. We employed the CRISPR-Cas13a system for simple, rapid, and direct detection of target miRNA. The amplification-free detection method enables specific recognition of single target miRNAs.^28^ We used AuNPs for detection probes. AuNPs exhibit strong scattering signals, providing the ability to quantify AuNPs by DF imaging. We formed magnetic-AuNP complexes connected with a DNA/RNA/DNA bridge that can be readily cleaved by activated Cas13a. Fig. 1A shows the process of miR-21-5p detection using the CRISPR/Cas13a-based system. In the presence of target miR-21-5p, the hybridization of the crRNA and miR-21-5p activates Cas13a proteins. The activated Cas13a protein then cleaves the bridge of MGNPs. Once the activated Cas13a proteins cleave the bridge, AuNPs are released and captured on a glass slide, while uncleaved complexes are easily removed by magnetic washing. The number of released AuNPs was counted by a custom-designed portable DF imaging system. The total assay is done in 30 min, including CRISPR/Cas13a reaction (10 min), AuNPs conjugation on a slide glass (15 min), and DF imaging (3 min).

We first prepared LwaCas13a and crRNA as described in our previous publication.^28^ To induce the expression of LwaCas13a, we utilized bacteria and subsequently purified the protein using a two-step process. The first step involved nickel–nitrilotriacetic acid (Ni-NTA) purification, followed by SUMO cleavage and a second round of Ni-NTA purification. We confirmed the successful purification of LwaCas13a by performing SDS-PAGE analysis and silver staining, which revealed a distinct band corresponding to the expected size of approximately 150 kDa, indicating the protein's high integrity (Fig. 1B). To synthesize crRNA for miR-21-5p, we employed *in vitro* transcription and confirmed the crRNA's integrity using denaturing PAGE and SYBR gold gel staining (Fig. 1C).

Secondly, we successfully synthesized and characterized the MGNPs. MGNPs were prepared by DNA hybridization between bridge and DNA linkers immobilized on the surface of the 60 nm AuNPs and 200 nm magnetic beads. The conjugation of MGNPs was validated by the dynamic light scattering (DLS) measurement (Fig. S1†). The mixture of AuNPs and magnetic beads without bridge linkers shows two distinct peaks in the DLS measurement. In adding bridge linkers, AuNPs and magnetic beads are successfully conjugated through the bridge linkers, showing a single peak in the DLS measurement (Fig. S1B†). In addition, MGNPs were further validated with scanning electron microscopy (SEM), which verifies the formation of the hybrid particles more clearly (Fig. 1D).

Next, we designed the portable DF imaging system to detect and quantify the released AuNPs (Fig. 1E and F). The DF imaging system has overall dimensions of 140 mm in length, 85 mm in width, and 250 mm in height, making it small enough to be classified as a portable system. The compact design of the DF imaging system could be achieved by side illumination of a light-emitting diode (LED) light. Unlike the conventional DF imaging system, the side illumination does not need to use a higher numerical aperture (NA) condenser, which makes the imaging system cost-effective, compact, and reliable for a portable system. The side-illuminated light coupled through the slide glass by total internal reflection could produce scattered light in the presence of AuNPs on the slide glass. This allows us to use a 10× objective lens and a USB camera to detect over 10 000 particles in a single image with a large field of view (1248 μm × 702 μm). Using the DF imaging system, we detected target miR-21-5p miRNAs by quantifying the number of released AuNPs (Fig. 1G). The developed DF imaging device showed promising features for practical applications, providing stable and reliable signal detection without the need for extensive calibration.

### Optimization of the CRISPR/Cas13a-assisted MGNP-DF assay

#### 1) Background noise reduction

**Fig. 2:**
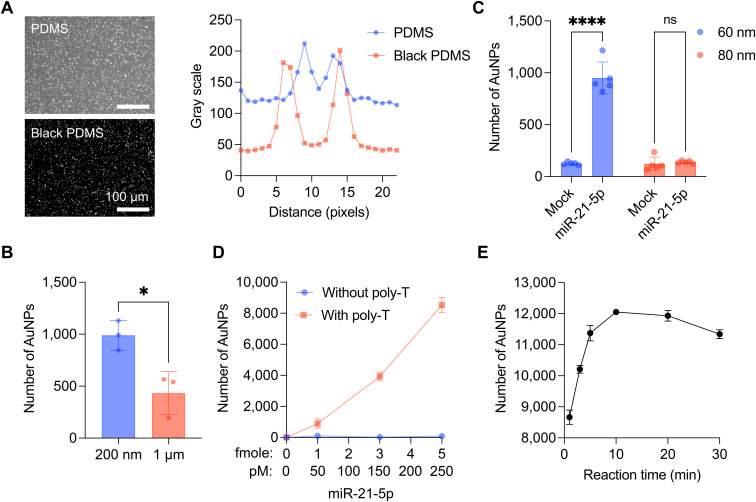
Optimization of the CRISPR/Cas13a-assisted MGNP-DF assay. A. DF images of released AuNPs with transparent and black PDMS (left) chambers. Comparison of plot profiles between PDMS and black PDMS chambers (right). Scale bar, 100 μm. B. Comparison of the size of magnetic beads. Magnetic beads with a 200 nm diameter showed a higher signal of AuNPs than magnetic beads with a 1 μm diameter. *P < 0.05 compared with 200 nm and 1 μm, as assessed by unpaired *t*-test. Bar graphs are shown as mean ± SD. C. Comparison of the size of AuNPs. AuNPs with a 60 nm diameter showed a higher signal than AuNPs with an 80 nm diameter by miR-21-5p-mediated Cas13a activation. ns, not significant; ****P < 0.0001 compared with the mock sample, as assessed by two-way ANOVA with Bonferroni's multiple comparisons tests. D. The number of AuNPs released from 3 different designs of MGNPs (1; linker only, 2; bridge without poly-T, 3; bridge with poly-T) depending on the amount of miR-21-5p. The reaction volume was 20 μl. Error bars are shown as mean ± SD. E. The number of AuNPs detached from magnetic beads depending on the Cas13a reaction time. Error bars are shown as mean ± SD.

To enhance the performance of our miRNA detection system, it was essential to minimize background noise in DF imaging, especially for an affordable, portable system. We thus explored the efficacy of black PDMS chambers in improving the signal-to-noise ratio in detecting scattering light from AuNPs. Given the nature of side illumination, background noise variability was anticipated based on the optical properties of the chamber. Comparative analysis between transparent PDMS (PDMS, Sylgard 184) and black PDMS (mixed PDMS with black ink, MG chemicals total ground carbon conductive coating, 838AR) demonstrated the superior noise reduction capabilities of black PDMS, resulting in a two-fold improvement in contrast (Fig. 2A).

#### 2) Size of magnetic beads and AuNPs

The sizes of both the magnetic beads and AuNPs were critical parameters in optimizing the CRISPR/Cas13a-assisted MGNP-DF assay. Initial comparisons between 200 nm- and 1 μm-sized magnetic beads revealed that MGNPs synthesized with 200 nm magnetic beads released approximately twice as many AuNPs post-CRISPR/Cas13a reaction (Fig. 2B). Similarly, different sizes of AuNPs were evaluated. AuNPs smaller than 50 nm were inadequately detected in the portable DF imaging system, showing a minimum size threshold (Fig. S2†). Comparative analysis between 60 nm and 80 nm AuNPs demonstrated significant signal differentiation with 60 nm AuNPs depending on Cas13a activity, likely due to different efficiencies of cleavages and AuNP release. Based on these results, we chose 200 nm magnetic beads and 60 nm AuNPs for MGNP synthesis (Fig. 2C).

#### 3) Bridge design

Three distinct designs of bridge linkers were evaluated for their efficacy in facilitating conjugation between MNPs and AuNPs (Fig. 2D and S3†) and cleavage by CRISPR/Cas13a. In the first design, we employed direct conjugation of AuNPs and magnetic beads through a single-stranded DNA/RNA/DNA linker with biotin and thiol functionalization for its simplicity. However, this design failed to exhibit cleavage by activated CRISPR/Cas13a proteins, rendering it unsuitable for the MGNP-DF assay. In the subsequent designs, we connected AuNPs and magnetic beads by sandwich hybridization with a single-stranded DNA/RNA/DNA linker (Fig. 1A). The DNA/RNA/DNA linkers hybridize to the DNAs functionalized on AuNPs and magnetic beads, respectively. Evaluation post-CRISPR/Cas13a cleavage showed the effectiveness of poly-T sequences on either side of the RNA sequence, enhancing bridge recognition by CRISPR/Cas13a (see Table S1† for the tested linker sequences).

#### 4) Reaction time

Determining the optimal reaction time for CRISPR/Cas13a was crucial for assay efficiency. Evaluation across reaction times ranging from 1 to 30 minutes revealed rapid AuNP detachment within the first 5 minutes, followed by saturation after 10 minutes. Consequently, 10 minutes was chosen as the optimized reaction time for CRISPR/Cas13a (Fig. 2E).

### Evaluation of detection sensitivity and specificity

**Fig. 3:**
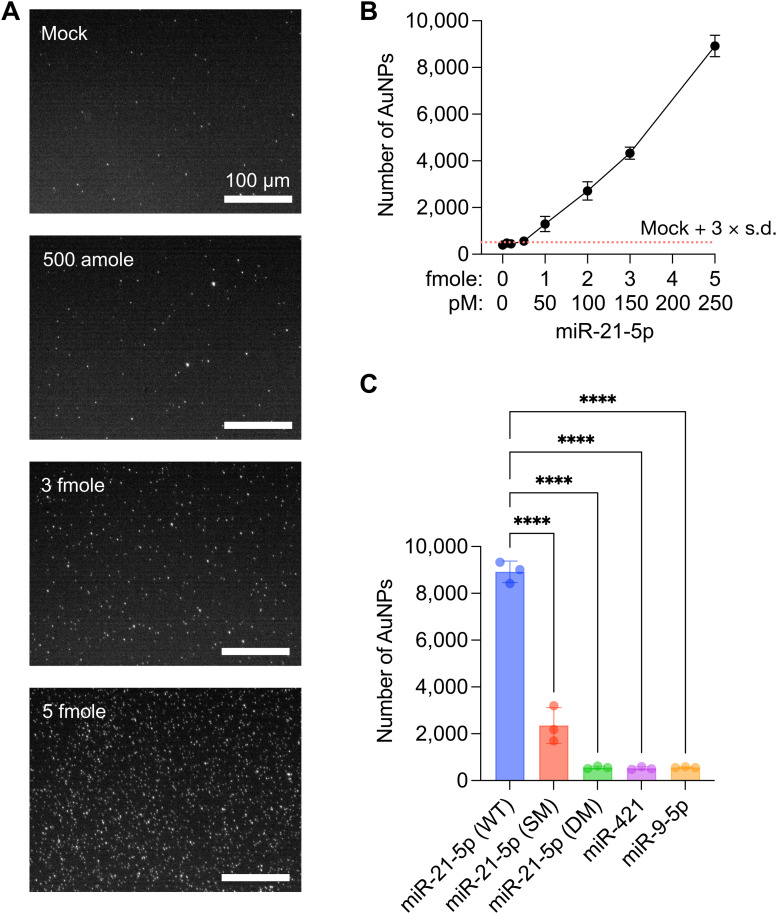
Evaluation of sensitivity and specificity of the CRISPR/Cas13a-assisted MGNP-DF assay. A and B. Measurement of released AuNPs by seven different concentrations of miR-21-5p. A. Representative zoomed-in images for the 4 different target miRNA amounts. Scale bar, 100 μm. B. Quantitative analysis for the numbers of released AuNPs. The dotted line indicates the number of mean + 3 × standard deviation (s.d.) of the mock sample. Error bars are shown as mean ± s.d. from three independent experiments. C. Detection specificity was evaluated using wildtype (WT), single-mismatch (SM), and double-mismatches (DM) of miR-21-5p, miR-421, and miR-9-5p. Bar graphs are shown as mean ± SD from the three experiments. ****P < 0.0001 compared miR-21-5p (WT) sample, as assessed by two-way ANOVA with Bonferroni's multiple comparisons test.

The sensitivity of the CRISPR/Cas13a-assisted MGNP-DF assay was evaluated through serial dilutions of miR-21-5p, employing MGNPs synthesized under optimized conditions (Fig. 3A). Titration experiments showed a LOD of 25 pM (500 attomoles in 20 μL) for miR-21-5p, as calculated by the concentration producing a signal greater than mean + 3 × standard deviation of the mock sample (Fig. 3B and S4†). It should be noted that this is a direct detection of target miRNA without any target sequence amplification (amplification-free detection). Notably, the quantity of released AuNPs from MGNPs exhibited a linear increase corresponding to the miR-21-5p concentration. To address the specificity of the developed assay, five distinct sequences were additionally employed: wildtype (WT), single-mismatch (SM), and double-mismatches (DM) of miR-21-5p, miR-421, and miR-9-5p (sequences were listed in Table S1†). As shown in Fig. 3C, DM, miR-421, and miR-9-5p failed to activate CRISPR/Cas13a and consequently did not induce the release of AuNPs from MGNPs. While the SM sequence exhibited a relatively higher number of released AuNPs than other sequences, it also demonstrated a significantly lower release than miR-21-5p (WT). Consequently, the MGNP-DF assay demonstrated the ability to differentiate at least a single-base mismatch with high specificity.

### Detection of miR-21-5p from breast cancer cell lines

**Fig. 4:**
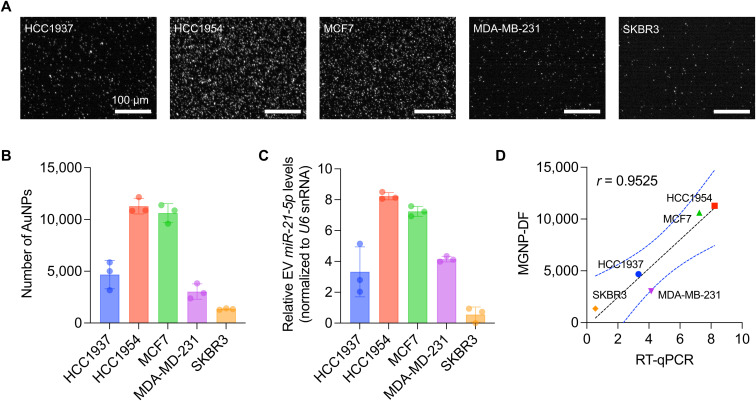
miR-21-5p detection from breast cancer cell lines. A. Representative zoomed-in DF images of released AuNPs by miR-21-5p-activated Cas13a. Scale bar, 100 μm. B. The number of released AuNPs in five different breast cancer cell lines. Bar graphs are shown as mean ± SD from the three independent experiments. C. Relative abundances of miR-21-5p, as analyzed by RT-qPCR. U6 snRNA levels were used as an internal control. Bar graphs are shown as mean ± SD from the three independent experiments. D. Dot plot for correlation coefficient. The number of released AuNPs from the CRISPR/Cas13a-assisted MGNP-DF assay and the relative abundance from RT-qPCR are indicated in the *y*- and *x*-axis, respectively. The black dashed line indicates the best linear fit. The two blue dashed lines indicate the 95% confidence interval.

The CRISPR/Cas13a-assisted MGNP-DF assay was employed to detect miR-21-5p levels in five distinct breast cancer cell lines, including HCC1937, HCC1954, MCF7, MDA-MB-231, and SKBR3 (Fig. 4A and B). We first isolated small RNAs from cell lysates and applied the assay to the isolated small RNAs. Notably, differential expression profiles were observed among the tested cell lines, with HCC1954 and MCF7 exhibiting high miR-21-5p expression levels, HCC1937 and MDA-MB-231 displaying intermediate expression, and SKBR3 demonstrating low expression (Fig. 4B). To validate the result obtained from our developed system, we performed RT-qPCR, which is considered the gold standard method for miRNA detection. 300 ng of small RNAs extracted from the five breast cancer cell lines were utilized for cDNA synthesis and PCR reactions (Fig. 4C). Notably, our developed system strongly correlated with the RT-qPCR results, as evidenced by a high Pearson correlation coefficient (*r* = 0.95, *p* = 0.0123, Fig. 4D). It should also be noted that our MGNP-DF assay required a 20-fold lower amount of RNA as input for detecting miR-21-5p without target amplification.

## Conclusions

The CRISPR/Cas13a-assisted MGNP-DF assay is a novel approach designed for simple, rapid, and accurate detection of target miRNAs, with a focus on point-of-care operation. The assay employs the CRISPR-Cas13a system for direct miRNA detection, eliminating the need for amplification. This method enables specific recognition of single target miRNAs, providing high sensitivity and specificity.

In this study, we prepared LwaCas13a and crRNA for miR-21-5p through bacterial expression and *in vitro* transcription, respectively. We then synthesized and characterized MGNPs, which are crucial components of the assay. Unlike other detection probes (*e.g.*, fluorophores), nanoparticles exhibit much stronger scattering signals, enabling accurate quantification of individual particles even using a portable system. This leads to high sensitivity without target amplification and accurate quantification, which is important to measure the relative expression of target miRNA between cancers and non-cancer cells. The DF imaging system was compact and efficient, providing stable and reliable signal detection without extensive calibration. The assay was optimized in several aspects, including background noise reduction, size of magnetic beads and AuNPs, bridge design, and reaction time. These optimizations improved the sensitivity and specificity of the assay, allowing for the detection of miR-21-5p at a LOD of the sub-femtomole range and the differentiation of at least single-base mismatches with high specificity. The validation with multiple breast cancer cell lines and comparison with the gold standard RT-qPCR shows the accuracy of our point-of-care system in quantifying target miRNA levels. For further applications, the system needs to be validated with clinical samples, which is lacking in the current study. We previously demonstrated that miRNA-21-5p detection in tumor-derived extracellular vesicles accurately identifies ovarian cancer patients from healthy controls.^28^ The developed MGNP-DF system could open up the possibility of conducting the test in point-of-care settings in clinics. In conclusion, the CRISPR/Cas13a-assisted MGNP-DF assay is a promising method for miRNA detection, offering simplicity, rapid analysis, accurate quantification, and point-of-care operation. Its high sensitivity, specificity, and reliability make it a valuable tool for miRNA research and clinical applications.

## Materials and methods

### Cell culture

The human breast cancer cell lines HCC1937, HCC1954, SKBR3, MCF7, and MDA-MB-231 were purchased from the American Type Culture Collection (ATCC). HCC1937, HCC1954, and SKBR3 cells were cultured in RPMI-1640 (Cytiva), and MCF7 and MDA-MB-231 were maintained in DMEM (Cytiva) at 37 °C in 5% CO_2_. All basal media were supplemented with 10% fetal bovine serum (FBS), 100 U mL^−1^ penicillin, and 100 μg mL^−1^ streptomycin (Millipore Sigma).

### Purification of LwaCas13a

We performed a purification of LwaCas13a, as previously reported.^28^ The plasmid DNA encoding LwaCas13a from Feng Zhang group (Addgene plasmid #90097; http://n2t.net/addgene:90097; RRID:Addgene_90097)^29^ were transformed into *E. coli* strain [Rosetta 2(DE3) pLysS (Millipore Sigma)] for bacterial expression. The LwaCas13a are purified by following cell lysis, 1st Ni-NTA purification, SUMO protease (ThermoFisher Scientific) treatment, and 2nd NI-NTA purification (Fig. 1B). The purified LwaCas13a were stored in the buffer (50 mM Tris-HCl, pH 7.5, 600 mM NaCl, and 2 mM DTT) with 5% glycerol and protease inhibitor until use at −80 °C.

### crRNA generation

The crRNA for detecting miR-21-5p was generated by *in vitro* transcription, as described previously.^28^ Briefly, the universal upper strand DNA, including the T7 promoter sequence, was annealed with the bottom strand DNA, including reverse complement T7 promoter and crRNA sequences. *In vitro* transcription was conducted with the annealed DNAs according to the manufacturer's description (Promega) and confirmed by 15% denaturing polyacrylamide gel electrophoresis (Fig. 1C). All DNA sequences used for crRNA generation are shown in Table S1.†

### Quantitative RT-qPCR

Small RNA isolation and RT-qPCR were performed as previously described.^28^ Briefly, small RNAs from the five different cells were isolated using the differential ethanol precipitation method. A total of 300 ng of small RNAs from HCC1937, HCC1954, MDA-MB-231, MCF7, and SKBR3 were used following polyadenylation and reverse transcription. The quantitative PCR was conducted using CFX Opus Real-Time PCR systems (Biorad), and the relative miRNA levels were determined using the –ΔCq values. U6 snRNA levels were used as an internal control. All DNA sequences used for RT-qPCR are shown in Table S1.†

### Preparation of magnetic beads with linkers

200 nm streptavidin-coated hydrophilic magnetic beads were purchased from Ocean Nanotech (SVO02000, 1 mg mL^−1^). We washed magnetic beads twice with a binding/wash buffer (pH = 7.5, 5 mM Tris-HCl, 1 M NaCl, and 0.5 mM EDTA). We added biotinylated MNP-linker (10 μM, 5 μL) and incubated the mixture for 2 hours at RT on a Hula mixer, followed by five-time washes with a wash buffer A (pH = 7.2, 20 mM HEPES, and 0.01% Tween 20) by magnetic separation to remove unbound MNP-linker. MNPs conjugated with MNP-linker were stored in a storage buffer (pH = 7.2, 20 mM HEPES, 0.1% BSA, and 0.01% Tween 20) at 4 °C. All linker sequences used for MNP conjugation are listed in Table S1.†

### Preparation of gold nanoparticles (AuNPs) with linkers

We used the previously described method to conjugate AuNP-linker to AuNPs.^30^ We mixed unconjugated 60 nm AuNPs (TedPella, 15709-20, 5.2 × 10^11^ particles mL^−1^; 50 μL), 50 mM HEPES 40 μL, and 200 μM BSA 10 μL, and incubated the mixture for 30 min at RT on a Hula mixer, followed by three-time washes with a wash buffer A (pH = 7.2, 20 mM HEPES, and 0.01% Tween-20) *via* centrifugation (3500 × *g*, 5 min). After washing, we added the TCEP-treated AuNP-linker (10 μM, 10 μL) into AuNP solution containing 300 mM KCl and incubated the mixture for 3 hours at RT on a Hula mixer. The AuNPs were washed with a wash buffer A (pH = 7.2, 20 mM HEPES, and 0.01% Tween-20) *via* centrifugation (3500 × *g*, 5 min, 3 times). AuNPs conjugated with AuNP-linker were stored in a storage buffer (pH = 7.2, 20 mM HEPES, 0.1% BSA, and 0.01% Tween-20) at 4 °C. All linker sequences used for AuNP conjugation are listed in Table S1.†

### Preparation of magnetic gold nanoparticles (MGNPs)

We prepared magnetic gold nanoparticles using 200 nm magnetic beads and 60 nm AuNPs connected with DNA/RNA/DNA bridges. We first added the DNA linker-conjugated magnetic beads (1 mg mL^−1^; 10 μL) and the DNA/RNA/DNA bridge (25 nM; 4 μL) in a hybridization buffer (pH = 7.2, 20 mM HEPES, 200 mM KCl, 0.1% BSA, and 0.01% Tween-20). We incubated the mixture for 30 min at 37 °C and then washed the magnetic beads five times by magnetic separation with a wash buffer B (pH = 7.2, 20 mM HEPES, 200 mM KCl, and 0.01% Tween-20) to remove the unbound bridge. The washed magnetic beads were resuspended in a hybridization buffer (pH = 7.2, 20 mM HEPES, 200 mM KCl, 0.1% BSA, and 0.01% Tween-20). We added the AuNPs conjugated with AuNP-linker (2.6 × 10^11^ particles mL^−1^; 20 μL) to the magnetic bead solution and incubated the mixture for 30 min at 37 °C. The MGNPs were formed after the incubation. The unbound AuNPs were removed by five-time washes with a wash buffer C (pH = 7.2, 20 mM HEPES, 5 mM KCl, and 0.01% Tween-20) by magnetic separation. The washed MGNPs were stored overnight at 4 °C on a Hula mixer before use.

### Preparation of a substrate coated with capture linkers

A black PDMS well was attached to a glass slide. We introduced 5 μL of streptavidin solution (Millipore Sigma, 50 μg mL^−1^ in 1× PBS) into the PDMS well and allowed it to incubate for 2 hours at RT. Following incubation, the PDMS well was rinsed with a binding/wash buffer (pH = 7.5, 5 mM Tris-HCl, 1 M NaCl, and 0.5 mM EDTA). Upon completion of the streptavidin coating, capture linkers (500 μM, dissolved in the binding/wash buffer) were added to the PDMS well and incubated for 1 hour at RT. After incubation, unbound capture linkers were removed with the binding/wash buffer. The substrate coated with capture linkers was then stored at 4 °C.

### CRISPR/Cas13a-assisted MGNP-DF assay

First, LwaCas13a (400 nM) and crRNA (200 nM) were mixed and incubated at RT for 15 min. We then mixed the aforementioned concentration of miR-21-5p or 15 ng of small RNA fraction from cell lines, Cas13a/crRNA complex, MGNPs, and reaction buffer (pH = 7.2, 20 mM HEPES, 60 mM KCl, 6 mM MgCl2, 0.1% BSA, and 0.05% Tween-20) with a final volume of 20 μL, and incubated at RT for 10 min on a Hula mixer. After 10 min incubation, the MGNPs were separated by a magnet. The supernatant was added to a black PDMS well attached to a slide glass and incubated for 15 min at 50 °C. The AuNPs contained in the supernatant were conjugated to the capture linkers coated on the slide glass. After 15 min incubation, we washed the black PDMS well with a wash buffer D (pH = 7.2, 20 mM HEPES, 60 mM KCl, 6 mM MgCl_2_, and 0.01% Tween-20) to remove the unbound AuNPs. The washed well was covered by a cover glass, and images for AuNPs were obtained using the portable dark-field microscope.

### Image analysis

The quantification of AuNPs was conducted using the ImageJ Comet plugin, with consistent detection parameters applied across all samples (approximate particle size: 4.0 pixels, intensity threshold: 3.0).

### Statistical analysis

All data were analyzed with GraphPad Prism (Version 10, GraphPad Software Inc., San Diego, CA, USA) and displayed as mean ± standard deviation. The unpaired *t*-test and two-way ANOVA with Boneferroni's multiple comparison tests were used to compare with control sets. Statistical significance was considered for values of *p* < 0.05.

## Data availability

Materials are available upon request by contacting the corresponding author.

## Author contributions

Conceptualization: Hyungsoon Im; data curation and formal analysis: Jae-Jun Kim, Jae-Sang Hong, Hyungsoon Im; funding acquisition: Hyungsoon Im; investigation: Jae-Jun Kim, Jae-Sang Hong, Hyunho Kim, Moonhyun Choi, Ursula Winter; methodology: Jae-Jun Kim, Jae-Sang Hong, Hyungsoon Im; resources and supervision: Hakho Lee, Hyungsoon Im; validation and visualization: Jae-Jun Kim, Jae-Sang Hong, Hyungsoon Im; writing – original draft: Jae-Jun Kim, Jae-Sang Hong, Hyungsoon Im; writing – review & editing: all authors.

## Conflicts of interest

H. I. is a scientific advisory board member of Nanopath and AITRICS and a consultant to Cellkey, Co. Ltd. H. I. receives research support from Canon Medical Research USA, Healcerion, and Noul. These activities have no relationship to the study presented here.

## Supplementary Material

SD-003-D4SD00081A-s001
