# Supplementary material for: CRISPR/Cas13a-assisted amplification-free miRNA biosensor via dark-field imaging and magnetic gold nanoparticles
Source: Sens Diagn. 2024 Jul 11;3(8):1310–8. doi: 10.1039/d4sd00081a (PMC11308380; doi:10.1039/d4sd00081a)
Supplement: SD-003-D4SD00081A-s001 [file SD-003-D4SD00081A-s001.pdf]

Electronic Supplementary Material (ESI) for Sensors & Diagnostics.
This journal is © The Royal Society of Chemistry 2024

Supporting Information

CRISPR/Cas13a-assisted amplification-free miRNA biosensor via dark-field imaging and magnetic gold nanoparticles

Jae-Jun Kim[1†], Jae-Sang Hong[1†], Hyunho Kim[1], Moonhyun Choi[1], Ursula Winter[1], Hakho Lee[1,2], Hyungsoon Im[1,2*]

[1] Center for Systems Biology, Massachusetts General Hospital, Boston, MA, 02114, USA
[2] Department of Radiology, Massachusetts General Hospital, Boston, MA, 02114, USA

[†]These authors contributed equally to the manuscript.

*Corresponding author:
Hyungsoon Im (im.hyungsoon@mgh.harvard.edu)

SUPPLEMENTARY FIGURES

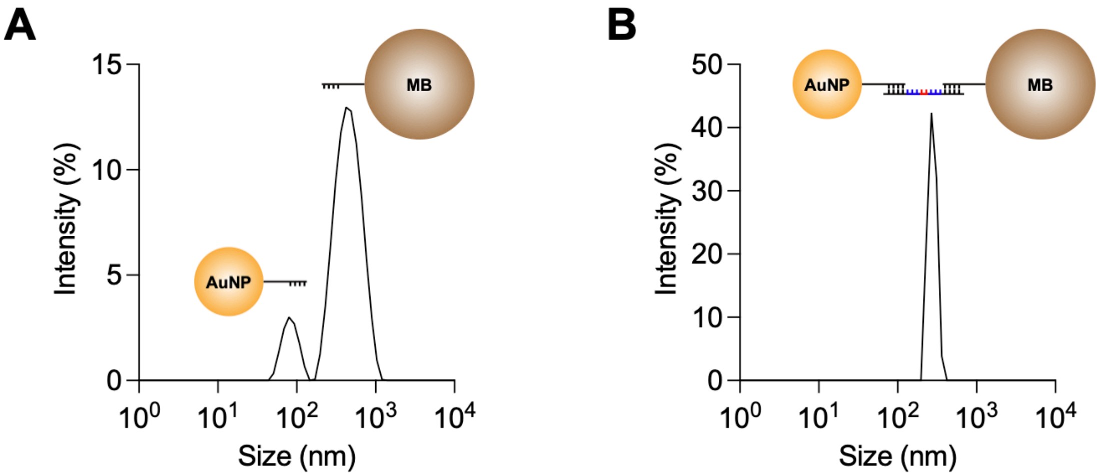

Figure S1. Dynamic Light Scattering (DLS) measurement results for mixture of magnetic beads (MBs) and gold nanoparticles (AuNPs) without (A) or with (B) a linker.

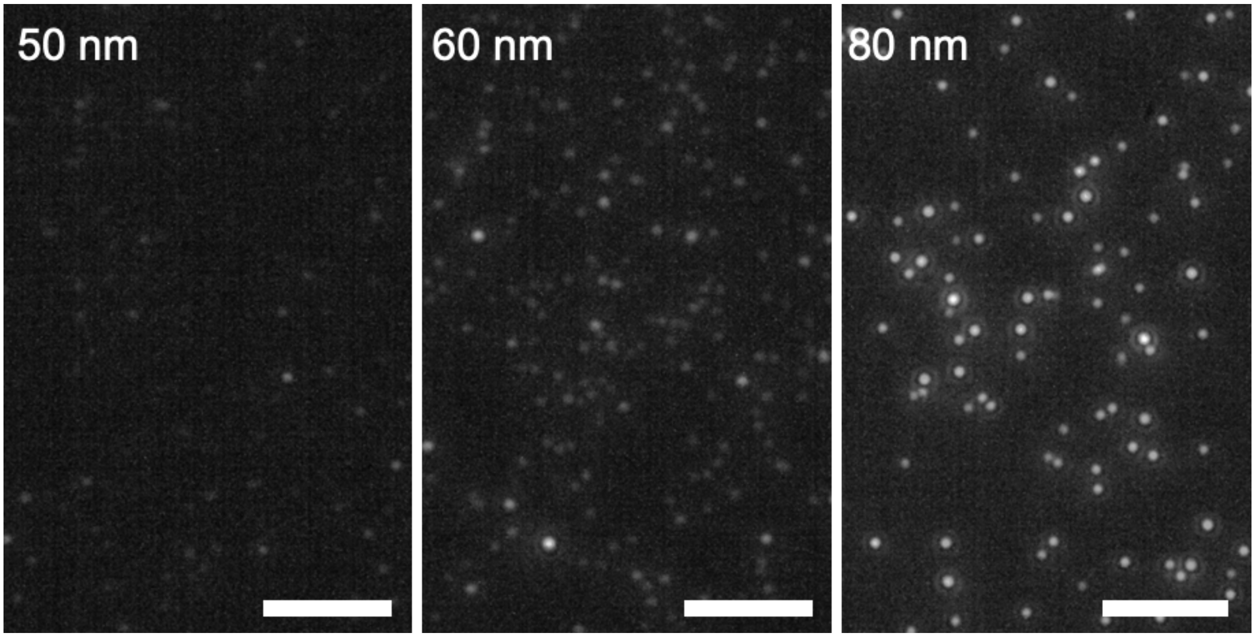

Figure S2. Dark-field (DF) images of gold nanoparticles (AuNPs) of different sizes (50 nm, 60 nm, and 80 nm) captured using a portable DF imaging system. Scale bar, 100 µm.

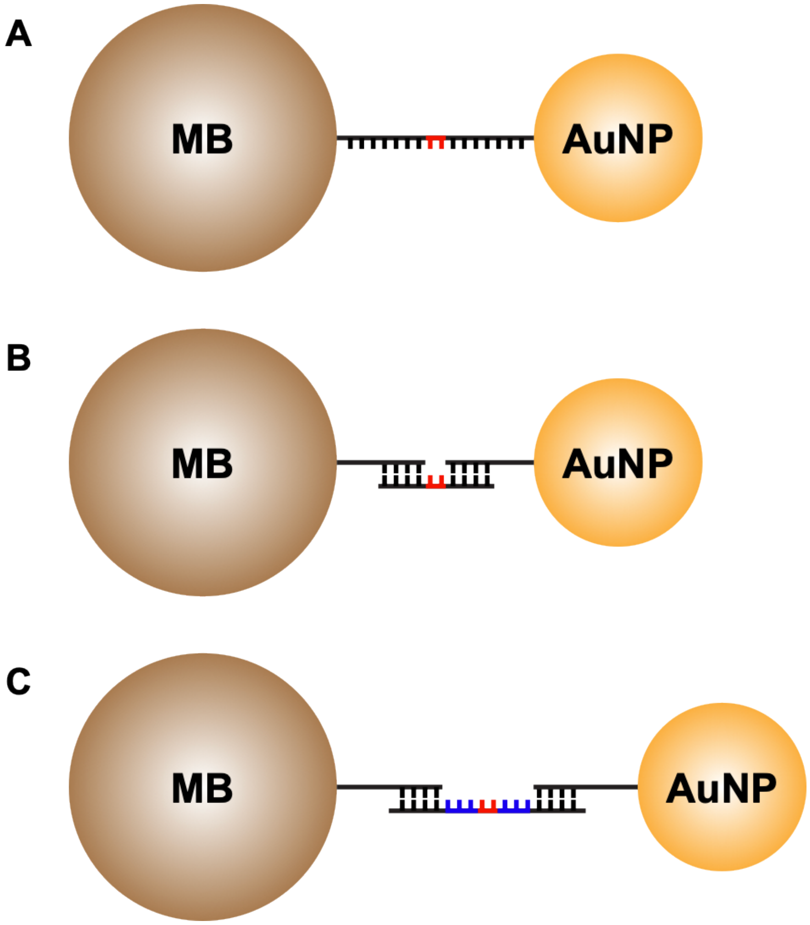

Figure S3. Hybridization strategies for linking magnetic beads (MBs) and gold nanoparticles (AuNPs) using nucleic acid linkers. A. Direct linking of MBs and AuNPs with a single-stranded DNA/RNA/DNA linker. **B-C.** Sandwich hybridization with bridges without (**B**) or with (**C**) poly-T. DNA and RNA sequences are denoted in black and red, respectively. Poly-T sequences are highlighted in blue.

A

Concentration	Mock	0.1 fmole 5 pM	0.2 fmole 10 pM	0.5 fmole 25 pM
Experiment 1	362.3	457.3	446.3	521.0
Experiment 2	387.0	434.3	408.0	573.3
Experiment 3	437.7	557.3	484.3	589.0
**Concentration**	**1 fmole 50 pM**	**2 fmole 100 pM**	**3 fmole 150 pM**	**5 fmole 250 pM**
Experiment 1	1073.7	3027.7	4625.3	9330.3
Experiment 2	1128.3	2264.0	4203.7	9011.7
Experiment 3	1674.0	2835.3	4156.0	8424.7

LOD threshold = mean + 3 × S.D.

= 395.7 + 3 × 38.4

= **510.9**

B

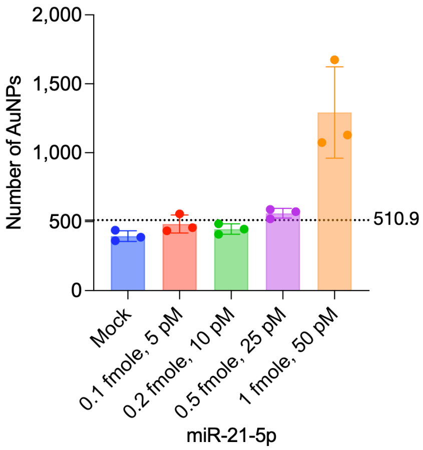

Figure S4. Calculation of the limit of the detection. A. Signal values for different concentrations of miR-21-5p and negative control (mock) to determine the limit of detection (LOD) threshold by the mean + 3 times the standard deviation. Based on the threshold value (510.9), we determined the LOD as 0.5 fmole (25 pM). **B.** Bar graph showing the signal values from different miR-21-5p concentrations and the LOD threshold value.

Supplemental Table S1. Sequences of oligomers used in this study.

Name	Sequence (5' to 3')[abc]
miR-21-5p-F	TAGCTTATCAGACTGATGTTGA
U6 snRNA-F	CAAATTCGTGAAGCGTTC CA
universal-R	AGGCAGTGGTATCAACGCAGA
miR-RT-adaptor	AGGCAGTGGTATCAACGCAGAGTACTTTTTTTTTTTTTTTTTTTTTTTTTTTTTTTVN
T7-univeral upper	TAATACGACTCACTATAG
T7-bottom miR-21-5p	TAGCTTATCAGACTGATGTTGAGTTTTAGTCCCCTTCATTTTTGGGGTGGTCTAAATCTATAGTGAGTCGTATTA
miR-21-5p (WT)	phospho-uagcuuaucagacugauguuga
miR-21-5p (SM)	phospho-uagcuuaucggacugauguuga
miR-21-5p (DM)	phospho-uugcuuaucggacugauguuga
miR-9-5p	phospho-ucuuugguuaucuagcuguauga
miR-421	phospho-aucaacagacauuaauugggcgc
DRD probe	FAM-TAuuGC-Quencher
Linker only	biotin-GATGGGCAGACAGGGTTAAGTTTTTTTTuuTAGGACTCGTGCAGCTAAGG-thiol
MB linker	biotin-TTTTTTTTTTGATGGGCAGACAGGGTTAAG
AuNP linker	TAGGACTCGTGCAGCTAAGGTTTTTTTTTT-thiol
Bridge without poly-T	CCTTAGCTGCACGAGTCCTATuuCCTTAACCCTGTCTGCCCATC
Bridge with poly-T	CCTTAGCTGCACGAGTCCTATTTTTTTTTuuTTTTTTTTCCTTAACCCTGTCTGCCCATC
Capture probe	CCTTAGCTGCACGAGTCCTATTTTTTTTTTTTTTT-biotin

[a] V = A, C, or G; N = A, C, G, or T

[b] Lower case letters denote RNA.

[c] Underline letters indicate mismatch.
